# Determinants of Peroxisome Membrane Dynamics

**DOI:** 10.3389/fphys.2022.834411

**Published:** 2022-02-03

**Authors:** Ruth E. Carmichael, Michael Schrader

**Affiliations:** College of Life and Environmental Sciences, Biosciences, University of Exeter, Exeter, United Kingdom

**Keywords:** peroxisomes, organelle dynamics, peroxin, PEX11, phospholipids, protein-lipid interactions

## Abstract

Organelles within the cell are highly dynamic entities, requiring dramatic morphological changes to support their function and maintenance. As a result, organelle membranes are also highly dynamic, adapting to a range of topologies as the organelle changes shape. In particular, peroxisomes—small, ubiquitous organelles involved in lipid metabolism and reactive oxygen species homeostasis—display a striking plasticity, for example, during the growth and division process by which they proliferate. During this process, the membrane of an existing peroxisome elongates to form a tubule, which then constricts and ultimately undergoes scission to generate new peroxisomes. Dysfunction of this plasticity leads to diseases with developmental and neurological phenotypes, highlighting the importance of peroxisome dynamics for healthy cell function. What controls the dynamics of peroxisomal membranes, and how this influences the dynamics of the peroxisomes themselves, is just beginning to be understood. In this review, we consider how the composition, biophysical properties, and protein-lipid interactions of peroxisomal membranes impacts on their dynamics, and in turn on the biogenesis and function of peroxisomes. In particular, we focus on the effect of the peroxin PEX11 on the peroxisome membrane, and its function as a major regulator of growth and division. Understanding the roles and regulation of peroxisomal membrane dynamics necessitates a multidisciplinary approach, encompassing knowledge across a range of model species and a number of fields including lipid biochemistry, biophysics and computational biology. Here, we present an integrated overview of our current understanding of the determinants of peroxisome membrane dynamics, and reflect on the outstanding questions still remaining to be solved.

## Introduction

**Figure 1:**
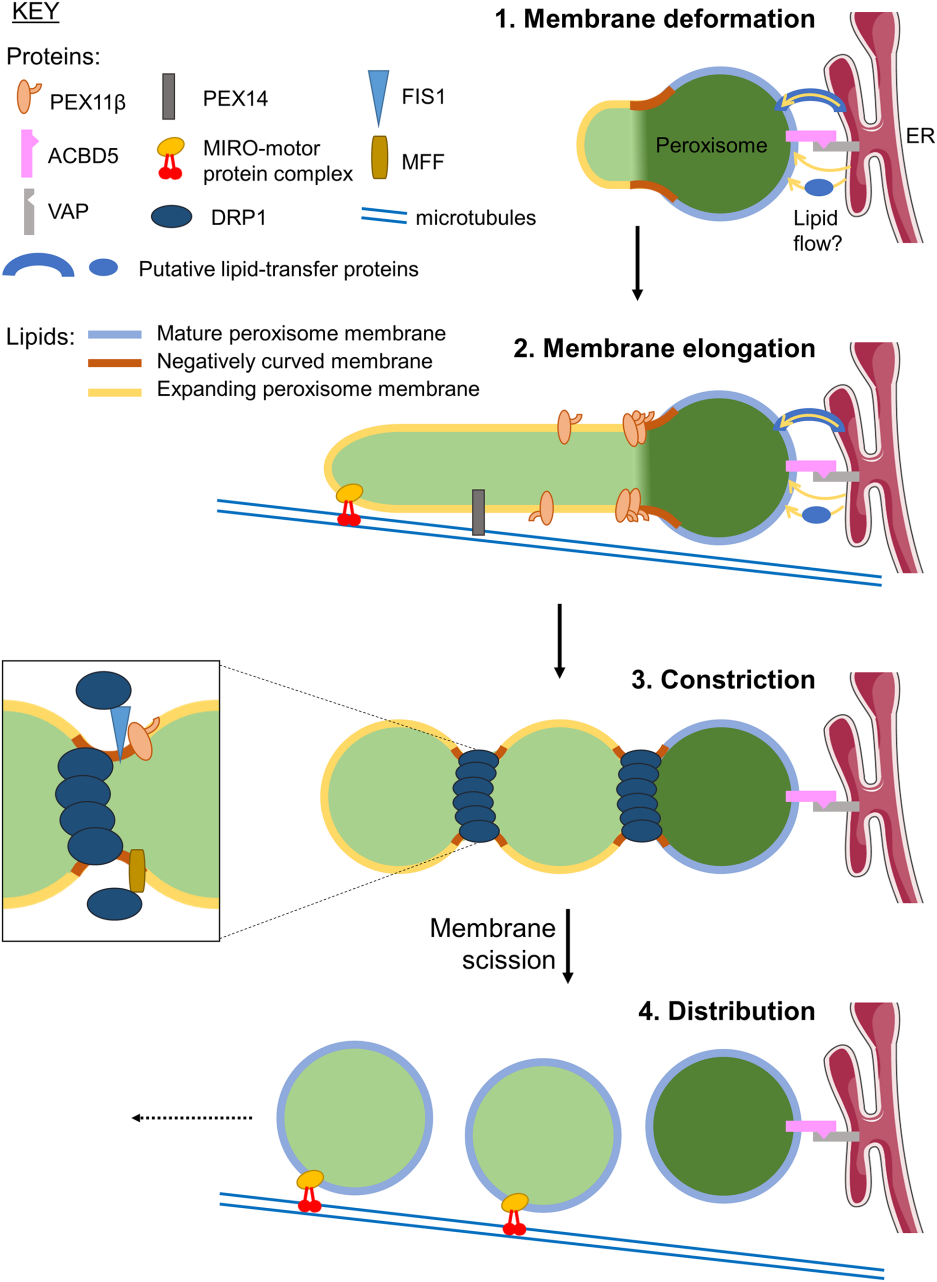
Growth and division of mammalian peroxisomes. Schematic showing the mechanism of peroxisome proliferation *via* a cycle of growth and division from pre-existing peroxisomes. Initially, the peroxisomal membrane deforms (1), with areas of membrane transitioning from positive to negative curvature to establish a protrusion. This protrusion then elongates, supported by lipid flow from the ER at ACBD5-VAP-mediated membrane contacts, *via* an unknown mechanism that may involve lipid transfer proteins. It is currently unclear whether the membrane composition of the protrusions, that will ultimately become the ‘daughter’ peroxisomal membrane, is the same or different to that of the ‘mother’ peroxisome. Elongation of the protrusion (2) requires PEX11β, and is facilitated by pulling forces along microtubules exerted by the peroxisomal MIRO1-motor protein complex. The elongated structures may be stabilised by binding to microtubules *via* the peroxisomal membrane protein (PMP) PEX14 (Passmore et al., 2020). The newly formed tubule is then constricted by a currently unknown mechanism allowing oligomerisation of the GTPase DRP1, forming a classical ‘beads-on-a-string’ morphology (3). DRP1 is recruited to the membrane by interacting with the adaptors FIS1 and MFF, which bind to PEX11β. DRP1-dependent GTP hydrolysis, facilitated by PEX11β, drives further constriction and ultimately membrane fission to generate multiple ‘daughter’ peroxisomes. These nascent peroxisomes import new matrix and membrane proteins to become fully functional, mature organelles, which are distributed through the cell along microtubules by the MIRO-motor protein complex (4). Elements of figure taken from Servier Medical Art (smart.servier.com).

Peroxisomes are multifunctional, oxidative organelles with key functions in cellular redox and lipid metabolism, which impact on human health and disease (Islinger et al., 2018). Peroxisomes are known for their remarkable plasticity, enabling them to efficiently adjust their shape/morphology, number, intracellular position, interorganelle interactions and metabolic functions in response to the changing needs of the cell or organism (Costello and Schrader, 2018). Particular focus has been on the dynamic processes that modulate and maintain organelle abundance by organelle formation (biogenesis). Peroxisomes can form from pre-existing organelles by processes of membrane growth and division (Schrader and Fahimi, 2006; Schrader et al., 2012b; Figure 1). This multi-step process involves remodelling of the peroxisomal membrane, membrane expansion/elongation (growth), membrane constriction and subsequent fission leading to the formation of new peroxisomes that import matrix and membrane proteins to retain functionality (for detailed reviews, see Nuttall et al., 2011; Costello and Schrader, 2018; Islinger et al., 2018). Recent findings revealed that peroxisomal membrane expansion depends on membrane contact sites with other organelles, in particular the endoplasmic reticulum (ER) in mammalian cells, which contribute to lipid transfer (Costello et al., 2017). In addition, tubular membrane extensions have been observed to participate in interactions with other organelles such as mitochondria, likely for metabolic and communicative purposes (Mathur et al., 2012; Kustatscher et al., 2019). Elongation of the peroxisomal membrane also depends on peroxisomal membrane proteins (PMPs) of the PEX11 family as well as on adaptor proteins, which link peroxisomes to motor proteins and the cytoskeleton in order to provide pulling forces to deform the peroxisomal membrane (Figure 1). To adopt this variety of morphologies, which range from spheres to membrane tubules with different diameters, the peroxisomal membrane needs to be highly dynamic, with extensive membrane remodelling. Although several key proteins involved in peroxisome dynamics and multiplication have been identified, little is known about alterations in peroxisomal lipid composition, biophysical properties of the membrane, and protein-lipid interactions, and their role in the regulation of these processes. The peroxisomal membrane needs to maintain its integrity at a range of both positive and negative curvatures, and it is likely that many, if not all stages of membrane expansion/growth and division are mediated by protein-lipid interactions.

Defects in peroxisomal membrane growth and division have been linked to new disorders with developmental defects, neurological abnormalities and loss of sensory functions. As some of the key peroxisome division proteins, such as mitochondrial fission factor (MFF) or dynamin-related protein 1 (DRP1) are shared with mitochondria (Schrader and Yoon, 2007; Costello et al., 2018), defects in these proteins affect both peroxisomal and mitochondrial morphology. Peroxisomes (and mitochondria) in MFF- or DRP1-deficient patient fibroblasts are highly elongated due to their inability to divide. The metabolic properties of the organelles are often not or only slightly compromised, highlighting the importance of membrane dynamics for human health and development (Passmore et al., 2020). MFF- or DRP1-deficient cells, therefore, represent valuable cellular models to investigate peroxisomal membrane dynamics.

In this review, we will outline what is currently known about the factors determining peroxisome membrane dynamics (including the phospholipid make-up, biophysical properties and protein-lipid interactions of the membrane) and the consequences of these for peroxisomal biogenesis and function, as well as considering the outstanding questions in the field.

### Peroxisome Membrane Lipid Composition

The membrane lipid composition of an organelle plays an important role in determining its dynamics and thus function, since the species of lipids present and their relative abundance will affect both the biophysical properties of the membrane, and its protein-lipid interactions. However, determining the membrane lipid profile of peroxisomes has proved challenging, due to the difficulty of isolating pure peroxisomal populations by subcellular fractionation.

#### Phospholipid Composition of the Peroxisomal Membrane

**Figure 2:**
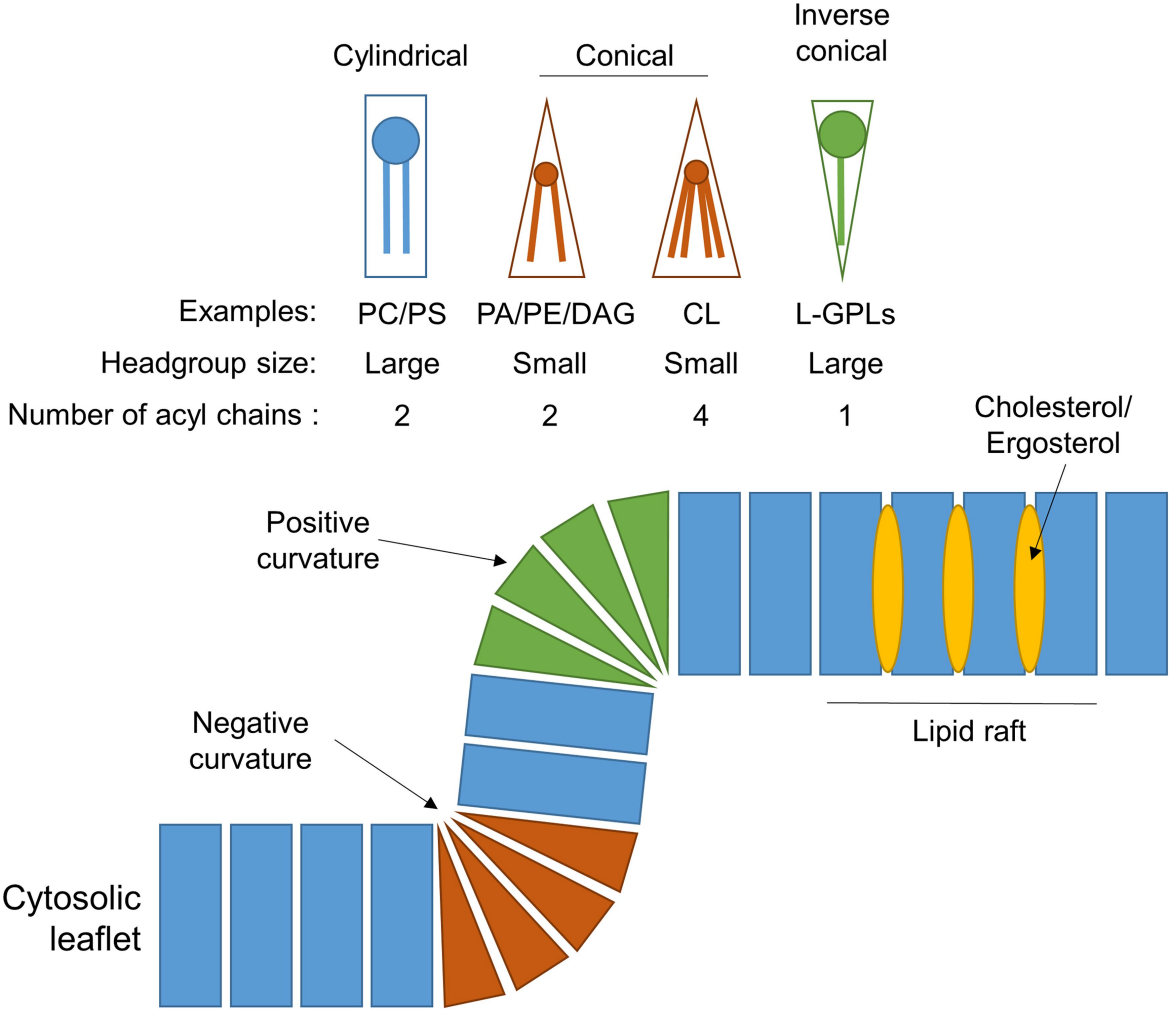
Phospholipid composition shapes the peroxisomal membrane. Schematic showing the different geometries of individual phospholipids, which depends on the size of their head group and/or number of acyl chains. The incorporation of these differently shaped phospholipids into a membrane leaflet will cause the membrane to adopt a positively-curved, negatively-curved, or planar topology, depending on the lipid species. The insertion of cholesterol (in mammals) or ergosterol (in fungi) into the hydrophobic region of the membrane generates lipid rafts with unique biophysical properties. For simplicity, only the outer leaflet of the peroxisomal membrane is shown. Examples: CL, cardiolipin; DAG, diacylglycerol; L-GPLs, lysoglycerophospholipids (e.g., LPA); PA, phosphatidic acid; PC, phosphatidylcholine; PE, phosphatidylethanolamine; and PS, phosphatidylserine.

Early studies in *Saccharomyces cerevisiae*, plants and rat liver determined that, like other intracellular eukaryotic membranes, phosphatidylcholine (PC) is the predominant lipid species found in peroxisomal membranes, comprising approximately 50% of the total phospholipids present. The remainder consists mostly of phosphatidylethanolamine (PE) and phosphatidylinositol (PI), with some phosphatidylserine (PS) in yeast and rat peroxisomal membranes (Donaldson et al., 1972; Fujiki et al., 1982; Hardeman et al., 1990; Chapman and Trelease, 1991; Zinser et al., 1991; Table 1). PE is notably enriched in both peroxisomal and mitochondrial membranes relative to other organelles, making up ~25%–30% of the total membrane lipids for these compartments. Since the small head-group of PE gives it a conical shape (Figure 2), its inclusion in predominantly PC-containing membranes imposes a curvature stress (van Meer et al., 2008), which could facilitate the dynamic processes of elongation, fission and (in the case of mitochondria) fusion that these organelles must undergo.

**Table 1:** Phospholipid composition of organelle membranes in different species and grown on different carbon sources, as percentage of total phospholipids.

	Rat liver^1^			*Saccharomyces cerevisiae* ^2^			*Pichia pastoris*					Plant (castor bean)^5^		
	PO	Mito	ER	PO	Mito	ER	PO^3^		Mito^3^		ER^4^	PO^*^	Mito	ER
							*MeOH*	*Oleic acid*	*MeOH*	*Oleic acid*				
**PC**	56.4	41.9	55.4	48.2	40.2	51.3	54.4	52.4	44.2	44.4	49.7	49.0	36.9	50.0
**PE**	29.6	29.0	18.4	22.9	26.5	33.4	27.6	26.6	34.7	37.2	24.5	31.4	30.9	26.6
**PS**	3.0	7.2	7.4	4.5	3.0	6.6	3.7	6.7	2.4	2.4	7.3	0	4.1	1.8
**PI**	4.7	3.7	10.5	15.8	14.6	7.5	6.3	6.1	3.2	2.0	9.0	6.1	14.3	18.9
**CL**	0	7.6	0	7.0	13.3	0.4	3.9	2.3	10.8	8.2	1.0	2.4	13.7	2.7

Phospholipids: PC, phosphatidylcholine; PE, phosphatidylethanolamine; PS, phosphatidylserine; PI, phosphatidylinositol; and CL, cardiolipin. Organelle membranes: PO, peroxisome; Mito, mitochondria; and ER, endoplasmic reticulum. Carbon sources: MeOH, methanol.

* Glyoxysomes (specialised peroxisomes housing key enzymes of the glyoxylate cycle) in plants.

1 (Hardeman et al., 1990).

2 (Zinser et al., 1991).

3 (Wriessnegger et al., 2007).

4 (Klug et al., 2014).

5 (Donaldson et al., 1972).

The yeast and plant, but not the mammalian, peroxisomal membrane also contains the dimeric phospholipid cardiolipin (CL) which, due to its small acidic head group and four acyl chains, has a conical structure, and can thus promote membrane curvature (Figure 2). It is most abundant in both yeast and mammalian mitochondrial membranes, where it plays an important role in mitochondrial fission by contributing to membrane bending (Paradies et al., 2019). It could have a similar function in the peroxisomal membrane, but its absence in mammals, which still have dynamic peroxisomes, suggest it is not an essential component for peroxisome membrane remodelling. Moreover, CL deficiency did not affect peroxisome biogenesis and proliferation in *S. cerevisiae* (Kawałek et al., 2016). Another difference between yeast and mammalian peroxisomes is that yeast peroxisomal membranes contain a higher proportion of PI as opposed to their mammalian counterparts (~16% vs. ~5%, respectively; Schrader et al., 2020), although PI content in the cytosolic leaflet of peroxisomes in intact mammalian cells is still enriched relative to the ER (Pemberton et al., 2020). Phosphorylated PI species (phosphoinositides) are important signalling molecules and protein interaction platforms that are differentially localised to the different cellular compartments, with distinct functions depending on the position and number of phosphate groups on the inositol head-group (Schink et al., 2016). Through radiolabelling and chromatographic separation, the predominant phosphoinositide species in rat hepatocytes have been identified as PI(4)P, PI(3,5)P_2_ and PI(4,5)P_2_ (Jeynov et al., 2006). PI(4,5)P_2_ is derived from phosphorylation of PI(5)P *in situ* by the kinase PI5P4K. In mammals, PI(4,5)P_2_ in the peroxisomal membrane is a key regulator of lipid metabolism, both promoting β-oxidation by facilitating lipid droplet transfer to peroxisomes (Ravi et al., 2021), as well as forming lysosome-peroxisome-ER contacts mediating cholesterol trafficking, through the tethering of lysosomal and ER synaptotagmin-related proteins directly to peroxisomal PI(4,5)P_2_ (Xiao et al., 2019). In *S. cerevisiae*, peroxisomes can also synthesise PI(3)P through phosphorylation of PI by the peroxisome-associated lipid kinase Vps34p, which is essential for signalling to induce regulated peroxisome degradation (pexophagy; Grunau et al., 2011). Since phosphoinositides are also implicated in regulating a number of dynamic membrane processes within the cell (including autophagy, endocytosis and membrane contact site formation) through recruitment of membrane remodelling proteins (Raiborg et al., 2016; Schink et al., 2016), it is tempting to speculate that phosphoinositides in the peroxisomal membrane could also play a similar role.

#### Origin of Phospholipids in the Peroxisomal Membrane

Since peroxisomes do not possess the necessary enzymes for phospholipid biosynthesis (Zinser et al., 1991), where do these membrane lipids come from? The lipid composition is fairly similar between peroxisome and ER membranes (Table 1), which could be explained by the *de novo* formation of peroxisomes as a consequence of fusion and maturation of ER-derived pre-peroxisomal vesicles (Kim et al., 2006). However, there is also evidence for a non-vesicular route to supply peroxisomal phospholipids, as yeast deficient in vesicular trafficking or *de novo* biogenesis could still transport lipids from the ER to peroxisomes, in an ATP-independent manner (Raychaudhuri and Prinz, 2008). Recent studies revealed that peroxisomal membrane expansion in mammals depends on peroxisome-ER contacts, which are mediated by the peroxisomal acyl-CoA binding domain (ACBD) protein ACBD5 and the ER-resident VAP [vesicle-associated membrane protein (VAMP) associated protein] proteins acting as membrane tethers (Costello et al., 2017; Hua et al., 2017; Figure 1). It is understood that membrane lipids are transferred to the peroxisomes at these contacts, in a non-vesicular fashion, to enable membrane elongation (Raychaudhuri and Prinz, 2008; Schrader et al., 2020). Several possible molecular mechanisms for lipid transfer across other membrane contact sites have been proposed, including: (1) movement of individual lipids within a hydrophobic pocket of a protein, which could be tethered to one or both membranes, or soluble (e.g., the oxysterol-binding protein related protein family; Raychaudhuri and Prinz, 2010; Boutry and Kim, 2021) or (2) bulk flow of lipids through a hydrophobic channel protein spanning the two organelles (e.g., the VPS13 protein family; Kumar et al., 2018). Notably, VPS13D has been observed to localise to ER-peroxisome contacts *via* the motor adaptor protein MIRO1 (Guillén-Samander et al., 2021), and is also required for normal peroxisome biogenesis (Baldwin et al., 2021). In conjunction with the rapid (and reversible) membrane expansion during the hyperelongation of peroxisomes in cells defective in peroxisome division (Castro et al., 2018; Passmore et al., 2020), we, therefore, speculate this ‘bulk flow’ model of lipid transfer may support peroxisome growth by transferring lipids from the ER to the peroxisomal membrane, although the exact mechanism remains to be elucidated.

#### Regulation and Functional Consequences of Peroxisome Membrane Lipid Composition

Changing the phospholipid make-up of a membrane has profound effects on its biophysical properties. In *S. cerevisiae*, mutants defective for PC synthesis have a lower PC/PE ratio in the peroxisomal membrane as opposed to wild-type, which leads to reduced fluidity of the membrane as assessed by fluorescence anisotropy (Flis et al., 2015). This in turn could compromise peroxisome biogenesis, as *S. cerevisiae* mutants with deficient PE synthesis (a necessary intermediate for PC synthesis) have a reduced number of peroxisomes compared to wild-type cells, which is rescued by supplying PC through an alternative biosynthetic pathway (Kawałek et al., 2016). Interestingly, studies in yeast have suggested that the lipid composition of the peroxisomal membrane can be altered in response to environmental or metabolic changes. Growing *Pichia pastoris* with methanol or oleic acid (a monounsaturated long-chain fatty acid, C18:1) as the sole carbon source, as opposed to glucose, induces proliferation of peroxisomes coupled with an increase in expression of enzymes required for methanol metabolism or fatty acid β-oxidation, respectively. Notably, the proportion of PS (Table 1) and dimethyl-PE in peroxisomal membranes was reduced in cells grown on methanol as opposed to oleic acid, whilst under oleic acid growth conditions, C18:1 concentration in the peroxisomal membrane was increased, suggesting it was incorporated into membrane lipids as well as being used as a carbon source (Wriessnegger et al., 2007). It is well established that the *S. cerevisiae* lipidome is significantly altered depending on the growth conditions (Klose et al., 2012), which may therefore change the cellular membrane lipid profile if these lipids are incorporated into phospholipids as well as being metabolised. The question remains as to whether these alterations to membrane lipid composition are just a passive consequence of metabolic changes induced by different carbon source availability generating different lipid species, or have also evolved as a way to integrate the environment with optimal peroxisome function. Supporting the latter idea, peroxisomal membranes from *S. cerevisiae* grown on oleic acid are significantly more disordered and thus more fluid than either mitochondrial or ER membranes under the same conditions (Reglinski et al., 2020). Since, under these conditions, peroxisomes are essential for cell growth and survival (being the sole organelle capable of fatty acid β-oxidation in yeast), it has been proposed that a specific change in peroxisomal membrane properties could be important for the metabolite exchange, protein sorting and membrane trafficking necessary to support survival on oleic acid as a sole carbon source. Additionally, shotgun lipidomics revealed significantly more PI in peroxisomal membranes as opposed to mitochondrial or ER membranes from yeast grown on oleic acid, suggesting phosphoinositide signalling may play a key role in peroxisome function under these growth conditions, for example, by regulating pexophagy (Reglinski et al., 2020). Since fungi cannot self-regulate temperature, the ability to responsively adapt membrane composition and flexibility may be necessary to maintain peroxisomal integrity over a wide range of environmental conditions. However, since this function is not required in mammals, it remains to be seen whether mammalian peroxisomes also change in terms of membrane fluidity in different contexts, for example, to remodel the peroxisomal membrane for growth and division (section The Connection Between Membrane Properties and Organelle Dynamics).

#### Lipid Domains Within the Peroxisomal Membrane

Notably, organelle membranes are not homogeneous, instead displaying high levels of lateral organisation, which means only considering the overall membrane lipid composition for an organelle may be overly simplistic. Phase separation, as a result of differential lipid interactions, leads to the formation of discrete subdomains within a phospholipid bilayer, possessing specific protein/lipid compositions and functional properties (Heberle and Feigenson, 2011; Levental et al., 2020). Due to the technical challenges of resolving such small areas of membranes, especially under physiological/live-cell conditions, there is limited evidence for the existence of lipid domains within the peroxisomal membrane. Typically, lipid rafts—subdomains enriched in cholesterol in mammals (or ergosterol in fungi; Figure 2)—can be separated from organelle membranes due to their insolubility in non-ionic detergents (Boukh-Viner et al., 2005). Non-ionic detergent treatment of isolated pre-peroxisomal vesicles from the yeast *Yarrowia lipolytica* liberates membrane domains enriched in ergosterol and ceramide (Boukh-Viner et al., 2005). In these immature vesicles, certain PMPs, including Pex1p and Pex6p, initially reside in these ergosterol- and ceramide-rich domains, but move laterally into ergosterol- and ceramide-poor regions. This is hypothesised to ‘prime’ the pre-peroxisomal vesicles to dock and possibly fuse, suggesting these membrane subdomains have a key functional role in regulating this proposed pathway of organelle maturation by fusion in *Y. lipolytica* (Boukh-Viner and Titorenko, 2006; Motley et al., 2015). Similarly, cholesterol-enriched lipid rafts have been isolated from peroxisomal membranes in human HepG2 cells, and showed differential enrichment of PMPs (Woudenberg et al., 2010). In particular, the fatty-acid transporter PMP70 (ABCD3) and the matrix protein import factor PEX14 were strongly associated with these domains, whereas another fatty-acid transporter, ALDP (adrenoleukodystrophy protein, ABCD1), was weakly associated, and the import factor PEX13 was depleted from these fractions altogether. Reduction of cellular cholesterol, leading to depletion of these lipid rafts, caused dissociation of the raft-associated PMPs, however, whilst PMP70 and PEX14 were still localised to the peroxisome membrane, ALDP trafficking was impaired, indicating different populations of lipid rafts with different properties may exist within the peroxisomal membrane (Woudenberg et al., 2010). Interestingly, cholesterol reduction also prevented the import of the matrix enzyme catalase into peroxisomes, suggesting peroxisome biogenesis may depend on these cholesterol-enriched lipid rafts.

As well as influencing the spatial organisation of proteins within a membrane to generate local functional ‘patches’ with unique environments, phase separation within a bilayer may also serve to regulate the interaction between organelles. Large intracellular ER-derived vesicles generated from hypotonic cell swelling undergo temperature-dependent phase separation into ordered and disordered lipid regions (King et al., 2020). Notably, intracellular contacts between these ER-derived vesicles and the plasma membrane, mitochondria and endosomes were observed to occur preferentially at ordered ER membrane subdomains, whereas lysosomes and peroxisomes formed contacts with the disordered regions, suggesting that the heterogeneous distribution of lipids in organelle membranes may serve to generate different interaction surfaces to regulate organelle contacts (King et al., 2020). Whether this is true of all organelles that form contacts, or is specific to the ER, remains to be seen.

### The Connection Between Membrane Properties and Organelle Dynamics

Organelles are highly dynamic entities, adopting different shapes as required for different aspects of their function—most obviously, in the case of peroxisomes, to divide and proliferate. The interdependent combination of physical forces acting on a bilayer and its biophysical properties (e.g., fluidity/rigidity, tension and degree of order) determine the dynamics of a membrane, and will therefore also impact on the dynamics of the organelle that the membrane forms the boundary of (Opaliński et al., 2011b).

#### Membrane Dynamics Determining Peroxisome Dynamics

The proliferation of mammalian peroxisomes following the growth and division model (Schrader and Fahimi, 2006) can be accurately recapitulated by computational modelling using only parameters relating to membrane dynamics. Here, peroxisome growth can be described by three processes requiring dynamic changes to the membrane: lipid flow into the peroxisome body; elongation of a membrane protrusion; and constriction and ultimately fission of the membrane (Castro et al., 2018; Passmore et al., 2020; Figure 1). However, little is known about how the biophysical properties of peroxisomal membranes facilitate or are modulated by these dynamic processes. Growth and division of peroxisomes requires extensive remodelling of the membrane, for example, the bilayer must adapt from positive to negative curvature during the constriction of the membrane prior to fission. Insights from the yeast *Y. lipolytica* suggest a putative model whereby local generation of the cone-shaped lipids phosphatidic acid (PA) and diacylglycerol (DAG), and depletion of the inverted cone-shaped lysophosphatidic acid (LPA), in the lumenal leaflet of the peroxisomal membrane serve to destabilise the lipid bilayer at constriction sites and drive the negative curvature required for fission (Boukh-Viner and Titorenko, 2006; Guo et al., 2007; Figure 2). However, whether this is sufficient to induce membrane curvature without additional protein forces, and whether this applies to multiple species, remains to be elucidated.

#### Forces Acting on Organelle Membranes

Forces that modulate membrane and organelle dynamics are better characterised for mitochondria, and may be extrapolatable to peroxisomes given the similarity of the division process for the two organelles (e.g., requiring the fission factors MFF, FIS1 and DRP1 in mammals; Schrader and Fahimi, 2006; Costello et al., 2018), though it is important to note that mitochondrial fission requires the scission of a double membrane, whereas peroxisome division only requires one membrane to divide. However, regardless of the organelle, fission requires not only topological changes, but also an increase in the elastic energy of the membrane, to overcome the unfavourable fission process (Mahecic et al., 2021). In mitochondrial and peroxisomal fission, the dynamin-related large GTPase DRP1 is recruited to the membrane, where it oligomerises into filaments that, upon GTP hydrolysis, curl up to encircle and constrict the organelle, providing the mechanical force to bend the membrane and drive scission (Koch et al., 2003; Kalia et al., 2018; Giacomello et al., 2020; Figure 1). Other constricting forces acting on the membrane are also supposed to contribute to mitochondrial fission, including ER-mitochondria contacts and the actin cytoskeleton (Friedman et al., 2011; Korobova et al., 2013). Indeed, in the case of peroxisomes, points of membrane constriction are still observed in cells where DRP1 is silenced, suggesting other forces are capable of deforming the membrane prior to fission (Koch et al., 2004).

Mechanical forces acting on mitochondria, such as collisions with bacteria, applied pressure from atomic force microscopy, or cell deformation across a patterned surface, can induce mitochondrial fission in a DRP1-dependent manner (Helle et al., 2017). Such mechanical stress induces tension in the membrane, which has recently been demonstrated to play a key role in governing mitochondrial fission (Mahecic et al., 2021). Exploiting newly-developed fluorescent sensors of membrane tension (Colom et al., 2018; Goujon et al., 2019) coupled with time-lapse super-resolution imaging, it was shown that DRP1-dependent constriction of the mitochondrial membrane was necessary but not sufficient to induce mitochondrial division, with successful fission events being associated with higher membrane tension generated by interactions with the cytoskeleton (Mahecic et al., 2021). Together, this implies that a combination of membrane bending (induced by constriction) and tension (induced by cytoskeletal forces) contribute to overcoming the energy barrier for fission by increasing the elastic energy stored in the mitochondrial membrane, with higher membrane tension therefore increasing the probability of a fission event at a constriction site (Feng and Kornmann, 2018; Mahecic et al., 2021). Since peroxisomal membrane tension can also be quantified with specific probes (Straková et al., 2020), it would be intriguing to test if this model also applies to peroxisome division.

### Protein-Lipid Interactions Regulating Peroxisome Membrane/Organelle Dynamics and Biogenesis

Organelle membranes are part of a complex environment with proteins both embedded within, and peripherally interacting with, the phospholipid bilayer. The interaction of soluble protein domains with membrane lipids can have profound impacts on the properties and dynamics of the membrane itself, and therefore organelle dynamics. In *S. cerevisiae* grown on oleic acid, peroxisomal membranes are more disordered than those of mitochondria and the ER, despite having equivalent levels of unsaturated lipids and ergosterol present, suggesting a different complement of interacting or integral proteins may be playing a role in determining membrane properties (Reglinski et al., 2020). Here, we will outline the function of proteins reported to interact with peroxisomal membrane lipids, and the role of these interactions in peroxisome proliferation and biogenesis, focussing on the PEX11 protein family as the best-characterised peroxisomal remodellers.

#### The Role of PEX11 in Peroxisome Membrane Dynamics

Proteins of the PEX11 family are key players in the control of peroxisome dynamics and proliferation. They represent a conserved group of membrane proteins in fungi, plants and mammals with functions in the regulation of peroxisome morphology, size and number (Thoms and Erdmann, 2005; Kiel et al., 2006; Aung et al., 2010; Chang et al., 2015; Schrader et al., 2016). Overexpression of PEX11 proteins often increases peroxisome abundance, whereas loss of PEX11 proteins reduces peroxisome numbers. Most species contain three PEX11 isoforms, which appear to vary in function. Pex11, Pex25, and Pex27 represent the Pex11 proteins in *S. cerevisiae* (Marshall et al., 1995; Rottensteiner et al., 2003; Huber et al., 2012); in mammals PEX11α, PEX11β and PEX11γ have been identified (Thoms and Erdmann, 2005; Schrader and Fahimi, 2006; Koch et al., 2010). There is currently no unifying nomenclature for the PEX11 proteins, which complicates comparison of isoforms and functions. For several PEX11 proteins, a role in the regulation of peroxisome number and size/shape has been revealed, however, other functions in fatty acid oxidation, as pore-forming proteins and in organelle interaction/contacts have also been described (van Roermund et al., 2000; Dulermo et al., 2015; Mattiazzi Ušaj et al., 2015; Mindthoff et al., 2016; Kustatscher et al., 2019; Wu et al., 2020).

Mammalian PEX11α, PEX11β and PEX11γ possess two transmembrane domains, which insert them into the peroxisomal membrane with their N- and C-termini exposed to the cytoplasm (Schrader et al., 1998b; Koch and Brocard, 2012; Bonekamp et al., 2013). Interestingly, PEX11β is removed from the peroxisomal membrane by post-fixation Triton-X 100 treatment (Schrader et al., 2012a; Bonekamp et al., 2013), indicating that the protein mainly interacts with membrane lipids rather than surrounding proteins. This does not apply to PEX11α and PEX11γ, suggesting different biochemical and functional properties. Several patients with a defect in PEX11β have now been identified, displaying a short stature, congenital cataracts, progressive hearing loss, and neurological abnormalities (Ebberink et al., 2012; Taylor et al., 2017; Tian et al., 2019). Patients with a defect in PEX11α or PEX11γ are currently unreported. Skin fibroblasts from PEX11β-deficient patients show reduced peroxisome numbers and altered peroxisome morphology indicative of a defect in peroxisome dynamics, whereas the metabolic functions of peroxisomes are not or only slightly altered underlining the importance of peroxisome plasticity for human health. Interestingly, altered peroxisome abundance in PEX11β-deficient epidermal cells caused abnormal mitosis and organelle inheritance affecting cell fate decisions (Asare et al., 2017). PEX11α expression in PEX11β-deficient fibroblasts did not restore the normal peroxisomal phenotype, whereas PEX11γ expression partially restored it further suggesting different functions of the PEX11 isoforms. In line with this, differences in the transcriptional regulation of PEX11α, PEX11β and PEX11γ have been reported (Azadi et al., 2020). In contrast to human patients, loss of PEX11β in knock-out mice is lethal and causes severe Zellweger-like symptoms (Li et al., 2002b). PEX11α knock-out mice are viable and show normal peroxisome abundance (Li et al., 2002a), but feeding a high-fat diet increased the rate of *de novo* lipogenesis, dyslipidaemia and obesity, decreased fatty acid β-oxidation and led to impaired physical activity and energy expenditure (Chen et al., 2019). Overall, these findings indicate that PEX11α and PEX11β differ in function, and may support a role for PEX11α in peroxisomal fatty acid metabolism rather than in peroxisome dynamics.

PEX11β is a membrane shaping protein that acts at multiple steps during the peroxisomal growth and division process, contributing to peroxisome multiplication/proliferation (Schrader et al., 2014, 2016; Figure 1). PEX11β promotes membrane expansion of pre-existing peroxisomes, which is a pre-requisite of peroxisomal division. Expression of PEX11β results in an elongation of the peroxisomal membrane, which is followed by membrane constriction and division resulting in multiple new peroxisomes (Delille et al., 2010; Schrader et al., 2016). Furthermore, lipid flow from the ER at ACBD5-VAP contact sites (Costello et al., 2017; Hua et al., 2017), and pulling forces along microtubules mediated by peroxisomal MIRO1, a membrane adaptor for the microtubule-dependent motor protein kinesin, also contribute to peroxisomal membrane expansion/elongation (Castro et al., 2018; Covill-Cooke et al., 2021; Figure 1). It has been revealed that besides membrane remodelling and expansion, PEX11β is also involved in the recruitment and assembly of the division machinery, as it interacts with the membrane adaptors FIS1 and MFF at peroxisomes (Koch et al., 2005; Kobayashi et al., 2007; Koch and Brocard, 2012; Itoyama et al., 2013). Those recruit DRP1 to the peroxisomal membrane, which mediates membrane scission (Koch et al., 2003, 2004; Li and Gould, 2003). PEX11β (and *Hansenula polymorpha* Pex11p) has been shown to stimulate the GTPase activity of DRP1, thus promoting DRP1 function (Williams et al., 2015).

The function of PEX11β and other PEX11 proteins in peroxisome membrane remodelling and elongation depends on the extreme N-terminal region, which is conserved amongst species and can adopt the structure of an amphipathic α-helix, consisting of hydrophobic and polar/positively charged residues arranged in a recurrent manner (Opaliński et al., 2011a). Upon insertion of this helix into the outer leaflet of the peroxisomal membrane, it is thought to cause membrane asymmetry and drive membrane bending/curvature, resulting in organelle tubulation (Opaliński et al., 2011a; Yoshida et al., 2015; Su et al., 2018). In line with this, deletions of the N-terminus or introduction of helix-breaking proline residues have been shown to inhibit peroxisome elongation (Kobayashi et al., 2007; Opaliński et al., 2011a; Bonekamp et al., 2013). PEX11 peptides containing the amphipathic helix from different species were shown to associate with liposomes *in vitro*, especially when negatively charged liposomes with a phospholipid content resembling that of yeast peroxisomal membranes (PC/PE/PI/PS/CL) were used (Opaliński et al., 2011a). Interaction of the PEX11 peptides with the negatively charged liposomes resulted in their tubulation (Opaliński et al., 2011a; Yoshida et al., 2015), whereas liposomes consisting solely of PC and PC/PE were not remodelled, probably due to a reduced affinity of the amphipathic helix for neutral membranes. Mutations affecting the hydrophobic surface of the amphipathic peptides or the helical structure abolished tubulation, whereas a gain-of-function mutant peptide with bulkier hydrophobic residues increased tubulation and the formation of tubules with a smaller diameter (10–15 nm instead of 40–50 nm).

Evidence also shows self-interaction/oligomerisation of PEX11 is important for membrane remodelling and membrane expansion. Oligomerisation of PEX11β depends on the N-terminal amphipathic region—N-terminal deletion or insertion of helix-breaking proline residues impaired oligomerisation and subsequent membrane elongation as well as the peroxisome-proliferating activity of PEX11β (Kobayashi et al., 2007; Bonekamp et al., 2013). Besides PEX11β homo-dimers, higher-order oligomers were also detected suggesting that PEX11β acts as a scaffold protein, which mediates membrane bending/remodelling and expansion through interaction with membrane lipids (Itoyama et al., 2012; Bonekamp et al., 2013). In this respect, larger PEX11β complexes might more strongly promote peroxisome elongation than smaller ones. Furthermore, molecular dynamics simulations showed that PEX11 peptides form linear aggregates on a model membrane (Su et al., 2018). Anionic lipids that compose the charged model membrane were observed to cluster around the peptide aggregates, likely due to peptide–lipid electrostatic interactions. Interestingly, PEX11β and yeast Pex11 proteins were reported to concentrate at specific regions of the peroxisomal membrane, e.g., prior to membrane tubulation and at constriction sites of elongated peroxisomes where they form patches (Schrader et al., 1998b; Kobayashi et al., 2007; Delille et al., 2010). Accumulation of Pex11p was also observed in *H. polymorpha* cells lacking Dnm1p (fungal DRP1 homologue), where Pex11p concentrated at the base of a peroxisomal tubule extending into the bud (Cepińska et al., 2011). The specific accumulation of PEX11 proteins through oligomerisation is supposed to present a starting point for membrane remodelling and assembly of the division machinery prior to fission (Itoyama et al., 2012; Bonekamp et al., 2013; Su et al., 2018). PEX11 oligomerisation and lipid interaction may thus contribute to the formation of lipid domains facilitating membrane curvature. As an anionic lipid, CL in the yeast peroxisomal membrane has the potential to be clustered by PEX11 oligomerisation and to contribute to membrane bending, though a block of CL synthesis has no effect on peroxisome formation in yeast (Kawałek et al., 2016).

PEX11β-induced peroxisomal division is impaired under lipid-free culture conditions, indicating that lipids are essential for peroxisomal membrane dynamics (Bonekamp et al., 2013). It is well known that peroxisomes are enlarged in size and reduced in number in fibroblasts from patients with defects in peroxisome biogenesis or peroxisomal β-oxidation enzymes (Santos et al., 1988; Chang et al., 1999; Funato et al., 2006; Itoyama et al., 2012). Interestingly, addition of docosahexaenoic acid (DHA, C22:6n-3), a major product of peroxisomal β-oxidation, to ACOX- or D-BF (*HSD17B4*)-deficient fibroblasts restored the normal peroxisome morphology and abundance (Itoyama et al., 2012). Other polyunsaturated fatty acids (PUFAs), such as eicosapentaenoic acid had a similar effect. DHA-induced peroxisome elongation and division was dependent on PEX11β and DRP1, and it was observed that DHA promoted PEX11β oligomerisation. PEX11β oligomerisation *in vitro* also correlated with the DHA content in liposomes (Itoyama et al., 2012). Peroxisomal membranes isolated from rat were reported to contain a high level of DHA, as approximately 25% of total PC and PE contain DHA (Yang et al., 2003). Membranes enriched in phospholipids containing PUFAs such as DHA show reduced rigidity, so also inherently promote bending (Harayama and Riezman, 2018). These findings suggest that DHA-containing phospholipids are required to remodel peroxisomal membranes. As peroxisomal β-oxidation is required for the synthesis of DHA in cooperation with the ER (Wanders et al., 2016) and as PUFAs stimulate peroxisome proliferation (Schrader et al., 1998a; Itoyama et al., 2012; Azadi et al., 2020), this may be a mechanism of integrating peroxisomal metabolism with peroxisome proliferation.

Tubular membrane extensions of peroxisomes were also observed to differ from the spherical mother peroxisomes they arise from. In addition to being morphologically distinct, spherical and tubular peroxisomal membrane compartments differ in the localisation of peroxisomal matrix and membrane proteins. Membrane proteins, such as PEX11β, FIS1 as well as the early peroxins PEX3, PEX16 and PEX19 involved in membrane protein targeting/insertion (see section PEX-Lipid Interactions Regulating Peroxisome Biogenesis) were observed to localise to the tubular domains induced by PEX11β expression, whereas matrix enzymes and membrane proteins with a metabolic function preferentially localised to the spherical domains (Delille et al., 2010). PEX11β-mediated peroxisome elongation also results in membrane reorganisation and the segregation of PEX11β from other peroxisomal proteins with accumulation in the tubular structures (Schrader et al., 1998b; Delille et al., 2010; Koch et al., 2010; Itoyama et al., 2012; Bonekamp et al., 2013). Redistribution of PMPs was also observed in yeast peroxisomes (Nagotu et al., 2008; Cepińska et al., 2011). It has been shown that membrane elongation initially results in the formation of a new membrane compartment, which subsequently constricts forming ‘beads on a string’-like structures and imports new matrix proteins (Delille et al., 2010). Membrane fission then results in the formation of new, spherical peroxisomes. The mother peroxisome appears to retain its matrix enzymes and certain membrane proteins indicating that peroxisomal growth and division is an asymmetric process (Huybrechts et al., 2009). How this is mediated and how the diffusion of PMPs is restricted is currently unknown. It is also unknown if there are differences in the lipid composition of the tubular and spherical membrane compartments, which could contribute to this differential protein distribution. Notably, ACBD5, which is involved in tethering of peroxisomes to the ER, localises preferentially to the spherical peroxisomes, which are associated with the ER (Costello et al., 2017; Bishop et al., 2019). Tubular peroxisomes in MFF-deficient fibroblasts were recently described to be composed of spherical membrane domains giving rise to highly elongated membrane extensions (Passmore et al., 2020). Immunofluorescence and fluorescent recovery after photobleaching experiments revealed that the spherical bodies represent mature, import-competent peroxisomes, whereas the tubular extensions comprise a pre-peroxisomal membrane compartment which has not yet fully acquired import competence for matrix proteins, or lacks the capability to retain them. Interestingly, the PMP PEX14 preferentially localised to the tubular extensions, potentially to stabilise them by linking the peroxisomal membrane to microtubules.

#### PEX-Lipid Interactions Regulating Peroxisome Biogenesis

**Figure 3:**
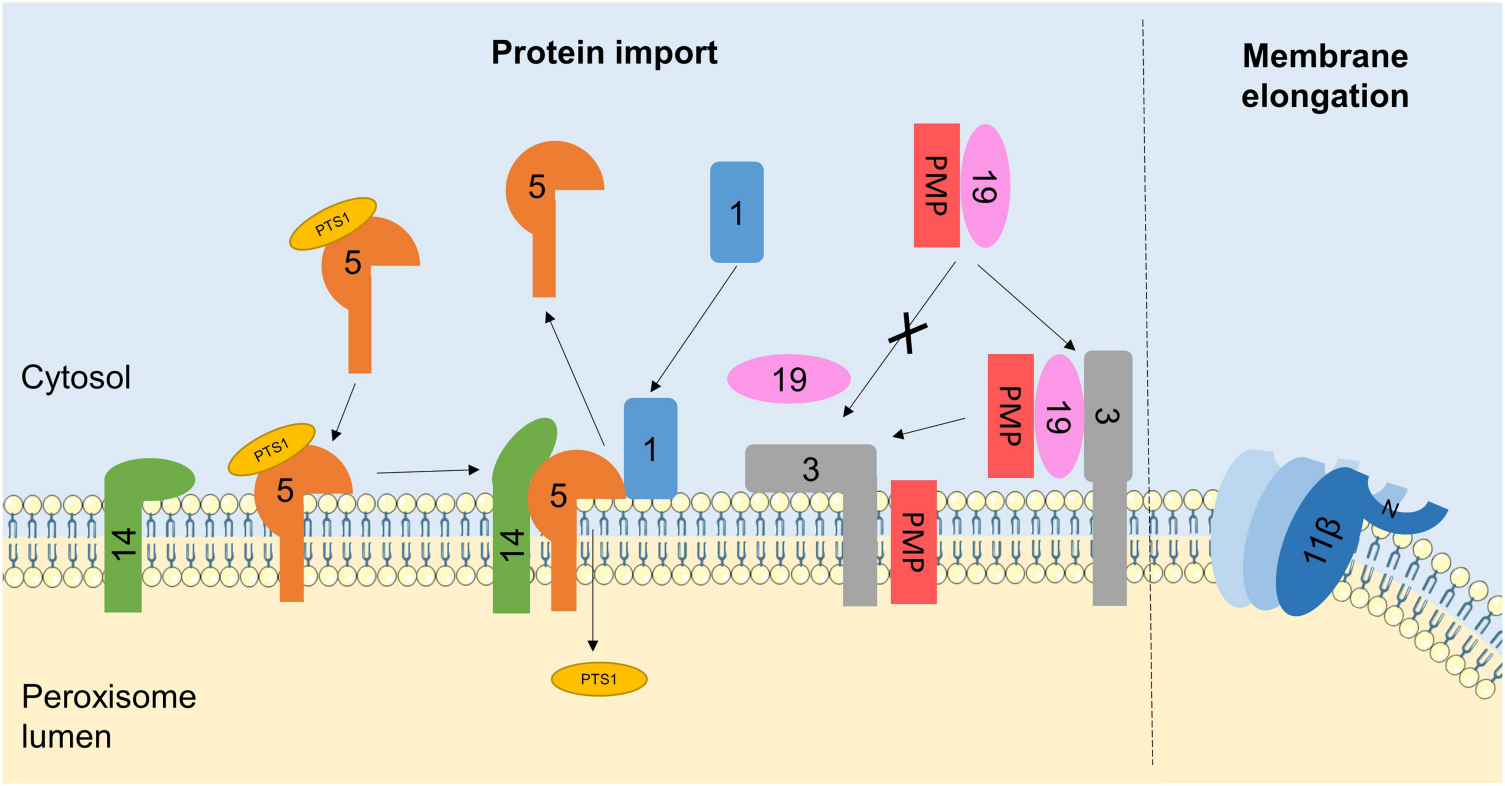
Interactions between PEX proteins and peroxisomal membrane lipids. Schematic of protein-lipid interactions regulating peroxisome biogenesis. For simplicity, PEX proteins are referred to by number only (with the PEX omitted). Interactions of PEX proteins with lipids in the peroxisomal membrane regulates PMP and matrix protein import. PEX1 recruitment to the membrane is facilitated by its binding to phosphoinositides. PEX1 is required for the recycling of the matrix protein receptor PEX5, which is itself stabilised by interactions with membrane lipids. The N-terminus of PEX14, part of the membrane docking complex for PEX5, can also bind to peroxisomal lipids, possibly to hide its PEX5-interacting face in the absence of PEX5, preventing non-specific binding to other cytosolic proteins. The PMP receptor PEX3 also interacts with lipid bilayers mimicking the peroxisomal membrane, which may serve to destabilise the membrane and release the chaperone PEX19, allowing for PMP insertion. Additionally, the interaction of PEX11β with the peroxisome membrane is important for its role in membrane elongation. Insertion of an amphipathic helix at the N-terminus of PEX11β into the lipid bilayer, as well as PEX11β oligomerisation, is required for membrane tubulation. Elements of figure taken from Servier Medical Art (smart.servier.com).

During peroxisome biogenesis, PEX proteins are essential for the insertion of newly-synthesised PMPs into the peroxisomal membrane, and the import of fully folded proteins from the cytosol to the matrix across the peroxisomal membrane, to ensure the growth and division process generates fully functional peroxisomes (Nuttall et al., 2011). Proteins destined for import into the peroxisomal matrix contain one of two peroxisomal targeting sequences: PTS1 at the C-terminus or PTS2 at the N-terminus. In mammals, PTS1-containing proteins are recognised by the cytosolic receptor PEX5, whereas PTS2-containing proteins bind a complex of PEX7 and a longer isoform of PEX5 (Lazarow, 2003). These receptor/cargo complexes dock to the PMP PEX14, allowing for cargo import across the peroxisomal membrane. The RING-domain proteins PEX2, PEX10 and PEX12 subsequently monoubiquitinate PEX5, signalling for its extraction from the membrane by a complex including the ATPases PEX1 and PEX6 (Fujiki et al., 2014; Farré et al., 2018). PMPs, on the other hand, are recognised in the cytosol by the receptor/chaperone PEX19, which is recruited to the peroxisomal membrane by PEX3/PEX16, allowing cargo insertion into the membrane (Jones et al., 2004; Figure 3). Notably, both matrix protein and PMP import require (1) recruitment of cytosolic PEX proteins to the peroxisome and (2) destabilisation of the membrane to allow proteins to be inserted or transported across, so as a result several PEX proteins have been reported to interact with membrane lipids directly (Figure 3).

PEX1 is an AAA-ATPase required for matrix protein import, by disassembling and recycling the matrix protein receptor PEX5 (Waterham and Ebberink, 2012; Figure 3). The structure of the N-terminal domain of mouse PEX1 was noted to be similar to that of another Type II AAA-ATPase known to bind phospholipids, VCP (Shiozawa et al., 2004). *In vitro* binding experiments demonstrated that the isolated N-terminal region of PEX1 displayed a broad specificity for binding to phosphoinositide species (especially the monophosphate forms: PI(3)P, PI(4)P and PI(5)P), with weak binding to PA and PS but no affinity for PE or PC (Shiozawa et al., 2006). Importantly, this N-terminal domain interacted with liposomes proportional to their phosphoinositide content, but showed non-specific binding kinetics, suggesting that PEX1 may be recruited from the cytosol to the peroxisomal membrane *via* its interaction with phosphoinositides (Shiozawa et al., 2006).

PEX5 itself also interacts with membrane lipids (Figure 3). PEX5 cycles between a cytosolic and membrane-associated state and interacts with the PMP PEX14 to deliver matrix proteins to the peroxisome for their import across the membrane. Using NMR, it was recently demonstrated that the disordered N-terminal domain of human PEX5 weakly interacts with membrane-mimicking lipid bicelles, which stabilises transient amphipathic helices in this region of the protein (Gaussmann et al., 2021). Similarly, the globular N-terminal domain of human PEX14 was also shown to interact weakly with peroxisome-like lipid bilayers, although this did not lead to any overall structural changes and was independent of its transmembrane domain. The association of PEX5 with the membrane does not significantly alter its binding affinity for PEX14 and the interaction of PEX14 with the membrane is readily outcompeted by its binding to PEX5, which occurs at partially overlapping sites (Gaussmann et al., 2021). Together, this suggests a possible model whereby the PEX14-phospholipid interaction partially hides the PEX5 binding site, preventing low-affinity non-specific interactions with other cytosolic proteins in the absence of PEX5, but being easily displaced when PEX5 is present to permit docking.

To be targeted to the peroxisomal membrane, PMPs form a complex with the cytosolic chaperone PEX19, which binds to the receptor PEX3 leading to PMP insertion into the peroxisomal membrane (Figure 3). PEX3 is believed to consist of a single transmembrane domain, and a large C-terminal region exposed to the cytosol (Soukupova et al., 1999). *In vitro*, this soluble C-terminal domain of human PEX3 (residues 34–373) aggregated in the presence of detergents, suggesting it interacts with amphipathic molecules. Accordingly, this region also interacted with lipids, in the form of small unilamellar vesicles mimicking the mammalian peroxisome membrane composition (PC/PE/PI/PS), leading to the hypothesis that this interaction may be important to destabilise the peroxisomal membrane to facilitate PMP insertion (Pinto et al., 2009). Interestingly, addition of recombinant PEX19 inhibited the PEX3-lipid interaction, suggesting binding of lipids and the PEX19-PMP complex to PEX3 is mutually exclusive, raising an intriguing possibility that reversible PEX3-lipid binding could act as a switch between the docking and insertion of PMPs (Pinto et al., 2009).

## Discussion

As illustrated here, the existence and persistence of functional peroxisomes within a cell is strongly dependent on the ability of the peroxisomal membrane to dynamically change its shape and properties, which is essential for growth and division *via* membrane elongation and fission, as well as matrix protein and PMP import. This membrane plasticity is generated through a number of factors, including phospholipid bilayer composition, biophysical forces, and protein-lipid interactions. It remains to be seen whether changes to the structure, shape and composition of the membrane is a driving force of organelle dynamics, or is required downstream of other processes to facilitate the necessary topological changes.

The importance of peroxisomal membrane dynamics for human health is increasingly being recognised, following the diagnosis of patients with mutations in proteins affecting the membrane remodelling of peroxisomes, including PEX11β (Ebberink et al., 2012; Taylor et al., 2017; Tian et al., 2019) and DRP1/MFF (which will also affect mitochondrial dynamics; Waterham et al., 2007; Passmore et al., 2020). These patients often present with neurological abnormalities, but importantly, the metabolic functions of the organelles are only marginally affected, indicating that the symptoms are a consequence of the loss of organelle membrane plasticity itself, which must therefore be essential for healthy cell function. Given that dysfunction of organelle division proteins, such as DRP1 are implicated in a number of disorders including neurodegeneration and cancer (Banerjee et al., 2022), it raises an intriguing possibility that reduced peroxisomal and/or mitochondrial dynamics could be a common pathological driver, and restoring membrane plasticity and dynamics could represent a promising therapeutic approach.

The contribution, regulation and interplay of the determinants of peroxisomal membrane dynamics outlined here, whilst currently less well characterised than for other organelles, is an area of active study at the interface of cell biology, lipid biochemistry, biophysics and computational biology. There are numerous outstanding questions in the field, including:

- How are phospholipids supplied to the peroxisomal membrane, and what is the role of membrane contact sites and lipid transfer proteins in this process?
- What are the biophysical properties of peroxisomal membranes, and do they play a role in peroxisomal dynamics?
- Is lipid composition variable in mammalian peroxisomal membranes, e.g., in response to environmental or metabolic changes?
- Do peroxisomal lipid properties regulate contact site formation?
- How do membrane lipid properties/interactions control matrix and membrane protein localisation?

Applying an interdisciplinary, integrated approach to answering these questions and elucidating the mechanisms and roles of peroxisomal membrane dynamics will provide vital new insights into healthy as well as pathophysiological peroxisomal function.

## Author Contributions

RC and MS conceived the project and wrote the manuscript. All authors contributed to the article and approved the submitted version.

## Funding

This work was supported by the Biotechnology and Biological Sciences Research Council (BBSRC) (BB/R016844/1, BB/T002255/1, to MS). RC is supported by BBSRC (BB/R016844/1). The research data supporting this publication are provided within this paper.

## Conflict of Interest

The authors declare that the research was conducted in the absence of any commercial or financial relationships that could be construed as a potential conflict of interest.

## Publisher’s Note

All claims expressed in this article are solely those of the authors and do not necessarily represent those of their affiliated organizations, or those of the publisher, the editors and the reviewers. Any product that may be evaluated in this article, or claim that may be made by its manufacturer, is not guaranteed or endorsed by the publisher.

## Abbreviations

ACBD5: Acyl-CoA binding domain protein 5
ALDP: Adrenoleukodystrophy protein
CL: Cardiolipin
DAG: Diacylglycerol
DHA: Docosahexaenoic acid
DRP1: Dynamin-related protein 1
ER: Endoplasmic reticulum
L-GPLs: Lysoglycerophospholipids
LPA: Lysophosphatidic acid
MFF: Mitochondrial fission factor
PA: Phosphatidic acid
PC: Phosphatidylcholine
PE: Phosphatidylethanolamine
PEX: Peroxin
PI: Phosphatidylinositol
PMP: Peroxisomal membrane protein
PS: Phosphatidylserine
PUFA: Polyunsaturated fatty acid
VAP: Vesicle-associated membrane protein (VAMP) associated protein
